# 3D-bioprinted microenvironments for sweat gland regeneration

**DOI:** 10.1093/burnst/tkab044

**Published:** 2022-01-21

**Authors:** Wei Song, Bin Yao, Dongzhen Zhu, Yijie Zhang, Zhao Li, Sha Huang, Xiaobing Fu

**Affiliations:** Research Center for Tissue Repair and Regeneration affiliated to the Medical Innovation Research Department, PLA General Hospital and PLA Medical College, 28 Fu Xing Road, Beijing 100853, P. R. China; PLA Key Laboratory of Tissue Repair and Regenerative Medicine and Beijing Key Research Laboratory of Skin Injury, Repair and Regeneration; Research Unit of Trauma Care, Tissue Repair and Regeneration, Chinese Academy of Medical Sciences, 2019RU051, 51 Fu Cheng Road, Beijing 100048, P. R. China; Research Center for Tissue Repair and Regeneration affiliated to the Medical Innovation Research Department, PLA General Hospital and PLA Medical College, 28 Fu Xing Road, Beijing 100853, P. R. China; PLA Key Laboratory of Tissue Repair and Regenerative Medicine and Beijing Key Research Laboratory of Skin Injury, Repair and Regeneration; Research Unit of Trauma Care, Tissue Repair and Regeneration, Chinese Academy of Medical Sciences, 2019RU051, 51 Fu Cheng Road, Beijing 100048, P. R. China; Department of Cardiac Surgery, and Department of Medical Sciences, Guangdong Cardiovascular Institute, Guangdong Provincial People's Hospital, Guangdong Academy of Medical Sciences, Guangzhou, Guangdong, 510100, China; Research Center for Tissue Repair and Regeneration affiliated to the Medical Innovation Research Department, PLA General Hospital and PLA Medical College, 28 Fu Xing Road, Beijing 100853, P. R. China; Research Center for Tissue Repair and Regeneration affiliated to the Medical Innovation Research Department, PLA General Hospital and PLA Medical College, 28 Fu Xing Road, Beijing 100853, P. R. China; Research Center for Tissue Repair and Regeneration affiliated to the Medical Innovation Research Department, PLA General Hospital and PLA Medical College, 28 Fu Xing Road, Beijing 100853, P. R. China; PLA Key Laboratory of Tissue Repair and Regenerative Medicine and Beijing Key Research Laboratory of Skin Injury, Repair and Regeneration; Research Unit of Trauma Care, Tissue Repair and Regeneration, Chinese Academy of Medical Sciences, 2019RU051, 51 Fu Cheng Road, Beijing 100048, P. R. China; Research Center for Tissue Repair and Regeneration affiliated to the Medical Innovation Research Department, PLA General Hospital and PLA Medical College, 28 Fu Xing Road, Beijing 100853, P. R. China; PLA Key Laboratory of Tissue Repair and Regenerative Medicine and Beijing Key Research Laboratory of Skin Injury, Repair and Regeneration; Research Unit of Trauma Care, Tissue Repair and Regeneration, Chinese Academy of Medical Sciences, 2019RU051, 51 Fu Cheng Road, Beijing 100048, P. R. China; Research Center for Tissue Repair and Regeneration affiliated to the Medical Innovation Research Department, PLA General Hospital and PLA Medical College, 28 Fu Xing Road, Beijing 100853, P. R. China; PLA Key Laboratory of Tissue Repair and Regenerative Medicine and Beijing Key Research Laboratory of Skin Injury, Repair and Regeneration; Research Unit of Trauma Care, Tissue Repair and Regeneration, Chinese Academy of Medical Sciences, 2019RU051, 51 Fu Cheng Road, Beijing 100048, P. R. China

**Keywords:** 3D bioprinting, Microenvironment, Sweat glands regeneration, Stem cells

## Abstract

The development of 3D bioprinting in recent years has provided new insights into the creation of *in vitro* microenvironments for promoting stem cell-based regeneration. Sweat glands (SGs) are mainly responsible for thermoregulation and are a highly differentiated organ with limited regenerative ability. Recent studies have focused on stem cell-based therapies as strategies for repairing SGs after deep dermal injury. In this review, we highlight the recent trend in 3D bioprinted native-like microenvironments and emphasize recent advances in functional SG regeneration using this technology. Furthermore, we discuss five possible regulatory mechanisms in terms of biochemical factors and structural and mechanical cues from 3D bioprinted microenvironments, as well as the most promising regulation from neighbor cells and the vascular microenvironment.

Highlights

- 3D-bioprinting technology shed light on regenerative medicine.
- Niche was essential for SG development and regeneration.
- 3D-bioprinted niche facilitated SG regeneration.
- Factors of 3D-bioprinted niche involved in SG regeneration.

## Background

Regenerative medicine is an interdisciplinary research field which spans the disciplines of tissue engineering, developmental cell biology, cellular therapeutics, gene therapy, biomaterials, chemical biology and nanotechnology [1]. To date, it has become increasingly recognized that the development and implementation of novel strategies can help in the repair, replacement or regeneration of damaged or diseased cells or tissues to restore their normal function. Among these numerous strategies, progress in the coupling of biomaterials to advance their use as scaffolds and matrices, as well as various advanced biomaterial-based integrated technologies, effectively promote regeneration both structurally and functionally or contribute to tissue healing [2,3].

Skin is located on the body surface and is relatively simple and highly organized in structure and function, therefore it is an optimal model for both basic and clinical regenerative research. It has long been known that sweat gland cells (SGCs), in particular, unlike other skin appendage cells, can participate in epithelialization during wound healing, although their own regeneration capability is very limited [4]. As an entire glandular tissue consisting of a functionally distinctive duct and secretory portions, regeneration of SGs seemed to be more difficult, but not inconceivable.

SGs are tiny and are distributed all over the body in humans to regulate body temperature. Dysfunction of SGs leads to hyperthermia which is linked to heat stroke and even death. An SG is a single tubular structure with a coiled secretory part below a straight duct. The secretory coil contains three distinct cell types: dark cells, clear cells and myoepithelial cells. Dark and clear cells are secretory cells, clear cells secrete water and electrolytes and dark cells secrete glycoproteins. Myoepithelial cells mechanically support the functional luminal structure. Mutations of ectodysplasin A, its receptor ectodysplasin A receptor and the adaptor protein EDAR-associated death domain cause abnormal development of SGs [5]. Mutation of Ca^2+^ release related genes *ITPR2, ORAI1* and *STIM1*, resulted in a marked reduction in sweat secretion despite normal SG development. The Foxa1–Best2 cascade was found to be involved in dark cells related to sweat secretion. Foxa1 mutant mice showed anhidrosis, while knockout of Best2 resulted in severe hypohidrosis [6]. Although cells of the secretory coil could respond to minor injury and proliferate to repair and cells of the duct could self-renew and replenish the epidermis and intraepidermal duct, severe burns caused failure of SG regeneration after the epidermis was restored [5].

We have proved that overexpression of some specific transcription factors could induce the transdifferentiation of epidermal cells and epidermal stem cells (ESCs) into SGCs [7,8], and bone marrow mesenchymal stem cells (BMSCs) and umbilical cord MSCs could differentiate into SGCs with treatment *in vitro*. However, the low efficiency of transdifferentiation, risk of exogenous gene introduction, long-term safety and the requirement for ethics permission have obstructed its clinical application. Hence it is urgent that a safe and more efficient strategy for SG regeneration be established.

The creation of microenvironments, often modeled on various stem cell niches that provide specific cues to manipulate cells, will likely be important in promoting optimal regenerative responses from therapeutic target cells [9]. Epithelial stem cells and SG-derived stem cells are both susceptible to niche regulation, and transplanting them to different body sites induced differentiation to other related tissues [4,10]. Our studies revealed that induced SGCs showed differential repair effects in different degrees of burn, which suggested that the difficulty of SG regeneration is strongly related to the destruction of their niche [8]. Additionally, adjusting the geometry and architecture of the tissue is of significant importance for guiding SG differentiation and morphogenesis. Therefore, reestablishing the SG niche *in vitro* paves an effective pathway to induce cell differentiation and tissue regeneration.

Conventional 3D biofabrication techniques such as scaffolding, microengineering and cell sheet engineering are limited in their capacity to fabricate complex tissue constructs to replicate biologically relevant tissues. 3D bioprinting offers great versatility to fabricate biomimetic, volumetric tissues that are structurally and functionally relevant. 3D bioprinting aims to generate and utilize biomimetic tissue or organ substitutes to replace, repair or augment those damaged by injuries or diseases [11]. Zopf *et al*. created a bioresorbable airway splint with laser-based 3D bioprinting to treat an infant with tracheobronchomalacia [12]; Laronda *et al*. created a bioprosthetic ovary using 3D printed microporous scaffolds to restore ovarian function in sterilized mice [13]; An *et al*. functionally reconstructed injured corpus cavernosa using 3D-printed biodegradable scaffolds seeded with hypoxia inducible factor 1 (HIF-1)-expressing stem cells [14]; Skardal *et al*. created single and integrated multi-organoid body-on-a-chip systems through 3D bioprinting for drug compound screening [15]; Deng created artificial dural onlay based on 3D printing technology to repair meningeal defects after brain trauma [16]; and Shokouhimehr *et al*. [17] and Sun *et al*. [18] created bone scaffold for jaw bone repair and damage-specific bone regeneration. Although emerging evolution in translational application of 3D bioprinting technology has been shown, the requirement for suitable bioinks, blood vessel construction and development of bioprinting technology still need to be tackled.

3D bioprinting technology has great potential for providing accurate and organized *in vitro* stimulation of the stem cell niche by recapitulating the highly organized tissue geometry of skin through spatiotemporal control of various types of bioinks and cells [3]. Native tissues are composed of various cell types and tissue morphogenesis is driven by cell–cell contact and biochemical signals. Structural and mechanical signals are important for SG differentiation and development, by which 3D bioprinted scaffold promotes self-organization. Moreover, blood vessels are important to functional tissues because the vascular network not only transports nutrients and oxygen, but also secretes growth factors to regulate adjacent cell behavior [9]. Furthermore, in terms of clinical application, delivering living cells with biocompatible materials, in sufficient numbers and within the right environment, with the use of 3D bioprinting, offers significant advantages [19].

**Figure 1.**
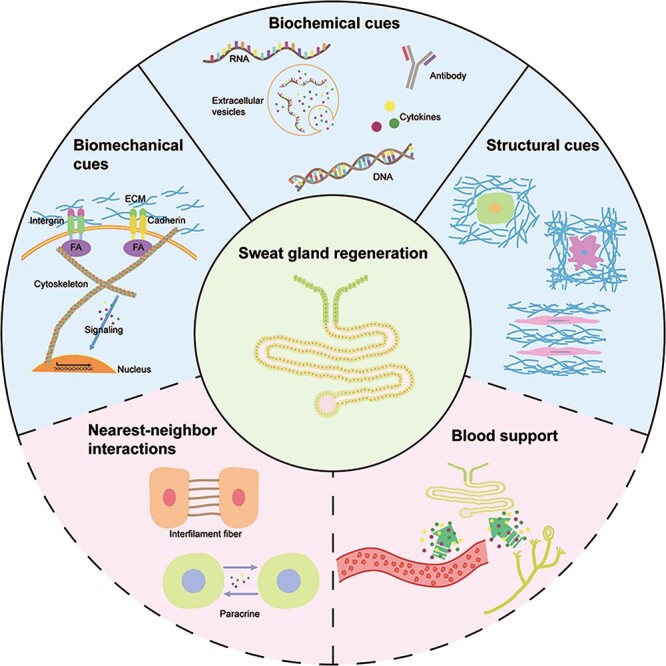
Microenvironmental factors involved in sweat gland regeneration. *ECM* extracellular matrix, *FA* f-actin, *DNA* deoxyribonucleic acid, *RNA* ribonucleic acid

In this article, we review the various factors involved in regeneration of SGs, including spatial structure, biochemical factors, mechanics, vascular niche and signals from neighbor cells (Figure 1).

## Review

### Research progress in SG regeneration

MSCs are the most widely used stem cells in the research of regenerative medicine because of their advantages, including low immunogenicity, they are less affected by the external factors of a large-scale wound, they can be stored in large numbers and are easy to access [20]. Li *et al.* observed that BMSCs could differentiate into SG-like cells directly when co-cultured with heat shock treated SGCs and promoted SG regeneration after transplanting into nude mice [21]. However, there were still some disadvantages such as trauma to the human body and a long inductive process. Compared with BMSCs, HUMSCs had higher proliferative activity and lower immunogenicity. Xu *et al.* showed that human umbilical mesenchymal stem cell (HUMSCs) could differentiated into SG-like cells and expressed SGCs markers when keratinocyte growth factor was added to the culture medium [22]. Liang *et al.* induced amniotic fluid MSCs differentiation into SG-like cells with conditioned medium of human SGCs, and found that sonic hedgehog played an important role in the induction [23]. Zhao *et al.* demonstrated that overexpression of nuclearfactor kappaB (NF-κB) and lymphoid enhanced factor 1 (Lef-1) in fibroblasts could reprogram fibroblasts into SG-like cells [24]. Furthermore, overexpression of Irf6 or foxc1 in epidermal cells induced differentiation into SGCs [7,8]. Although the above methods successfully induced regeneration of SGCs, clinical application was still restricted by their limited predictive performance *in vivo*.

### The role of niche in SG development

Differentiation of cells on the surface of the ectoderm led to the development of the mammalian epidermis, including the stratification and the formation of skin appendages such as SGs, hair follicles and sebaceous glands [25]. The niche was mainly composed of neighbor cells, glycosaminoglycans, adhesion proteins, structural proteins and soluble factors [26]. Plenty of studies have shown that niche plays an important role in regulating cell localization, behavior and differentiation [4,10]. In order to challenge the impact of niche on cell fate, Ferraris *et al.* co-cultured corneal epithelial stem cells with embryonic mouse foot dermis, upper lip dermis and dorsal dermis. The results showed that the cells co-cultured with foot dermis expressed SG-specific keratin (K9) and the cells co-cultured with back dermis formed hair follicle morphology and expressed hair follicle-specific keratin (K15), while the cells co-cultured with the upper lip dermis differentiated into epidermis with a multi-layer structure [10]. In addition, Lu *et al.* transplanted SG progenitor cells into a mouse mammary gland fat pad, and found that they formed branches like mammary glands with decreased expression of ATP1a1 and increased expression of milk protein. SG progenitor cells grafted into mouse back skin could form stratified epidermis. However, when SG progenitor cells were transplanted into a mouse shoulder fat pad, they formed a curly tubular shape and expressed SG-specific keratin (K18) [4]. Collectively, microenvironmental factors played an essential role in fate specification.

In patients with extensive trauma, although the skin injury could be repaired after skin transplantation, SGs could not be regenerated [27]. Our research showed that the damage of niche might be responsible for loss of SGs [8,27]. In mice, epithelial stem cells differentiated into SGs in the paw pad while they differentiated into hair follicles and sebaceous glands in the back [27]. This specific distribution of skin appendages provided a great animal model to study the impact of niche on cell behavior.

### Progress of 3D bioprinting technology in regenerative medicine

3D bioprinting is a comprehensive technology that integrates computer-aided design, computer-aided manufacturing, computer numerical control, precision drive, biomaterials, cell biology and molecular biology. 3D bioprinting not only provides a new clinical method for tissue repair, but also an exciting research tool for regenerative medicine, tissue engineering, stem cells, cancer and other research fields [28]. Pati *et al.* successfully induced adipose MSCs to express the specific markers of cardiomyocytes and chondrocytes by adding extracellular matrix (ECM) of myocardial tissue and cartilage into bioink and constructing a 3D structure with 3D bioprinting technology [29]. Lee *et al*. induced endogenous MSCs to differentiate into menisci *in vivo* by constructing 3D printing scaffolds that could continuously release a variety of growth factors, and repaired meniscus damage in sheep [30]. Miller *et al*. utilized stereolithography to print out a functional vascular system and simulate oxygen exchange in the alveolar structure [31]. Feinberg *et al.* used freeform reversible embedding of suspended hydrogels to print collagen through 3D bioprinting to control the composition and structure accurately, and constructed a heart [32]. The above studies have fully proved that 3D bioprinting has great potential in simulating niche to induce stem cell differentiation and the reconstruction of organs *in vitro*.

Actually, there are no effective clinical treatments for SG regeneration at present. Firstly, the regenerative capability of SG is weak and the maintenance of morphology and function of SG significantly depends on its niche. Secondly, in a severe burn or wound, the niche of SG is destroyed completely. The structure of skin appendages like SGs is tiny and subtle, which is why SGs are difficult to isolate and purify, blocking the progress of artificial skin. However, induced SG with the use of 3D bioprinted niche and MSCs could greatly advance clinical treatment. The technique not only provides numerous and safe source of SGCs for transplantation, but also the artificial niche supports the maintenance of regenerated SG to promote functional regeneration. Therefore, 3D printing is important and necessary for SG regeneration.

### 3D bioprinted niche facilitated SG regeneration

#### 3D bioprinted niche induced ESC differentiation into SGCs

**Figure 2.**
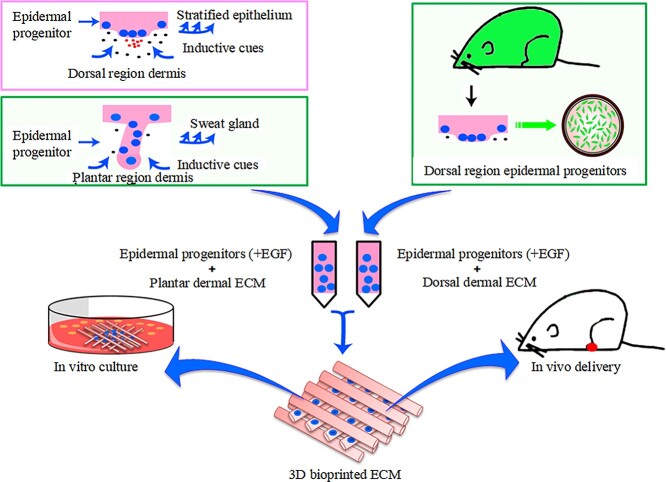
3D bioprinted niche induces functional sweat gland regeneration. *EGF* epidermal growth factor, *ECM* extracellular matrix

ESCs were considered as seed cells for SG regeneration as SGs are derived from embryonic ESCs. Given that dermal components and growth factors in ECM were vital for epithelial cell-fate determination, homogenate of mouse paw pad and epidermal growth factor (EGF) were added into a mixture of gelatin and sodium alginate as bioink to print with the set model and construct *in vitro* niche [33,34]. SG-like cells were observed after 14 days of culture and transplanting the substitute SG-like cells into a burn model could partially repair functional SG tissue. As niche is complex and multi-dimensional, a single EGF could not induce cell differentiation, and the efficiency of cell differentiation in conventional culture methods was much lower than in a 3D environment. Tissue homogenates provide complex inducible growth factors and biomaterial scaffolds provide structural space and attachment sites for cell differentiation, which made it possible for 3D printing models to accurately induce directional differentiation of cells and promote cell proliferation and matrix secretion. Surprisingly, the efficiency of cell differentiation reached >50% after 2 weeks of culture (Figure 2) [34]. In terms of safety, it is not necessary to consider tumor formation and exogenous gene intake. In a word, the strategy was safer than previous methods for avoiding tumor formation induced by foreign gene intake and it showed excellent potential for clinical application due to the short induction time and high inductive efficiency.

**Figure 3.**
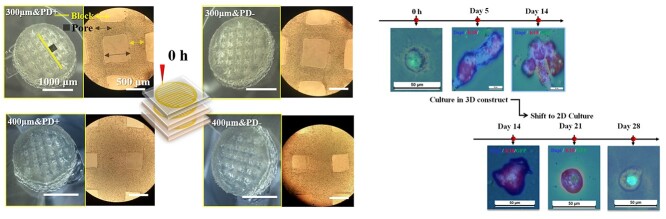
3D scaffolds with different structures and their influences on cell behavior and tissue morphogenesis. *PD* plantar dermis

Cell behaviors such as cell proliferation, migration and gene expression depend on the geometry of niche, which fosters cell cytoskeletal tension and provides cell adhesion [35]. Researchers have demonstrated that the apoptosis rate of cells increased in a smaller space but decreased in a larger cell space because the nucleus would become larger resulting in increased chromatin depolymerization and DNA synthesis and cell proliferation [36,37]. MSCs tended to be adipogenic when they were small and osteogenic when large [38]. Therefore, we further studied the role of structural factors in SGC induction [39]. Two printing nozzles with diameters of 300 and 400 μm, respectively, were used to construct 3D scaffold with different pore diameter and thread width. There were no significant differences in cell viability and proliferative capacity between the two groups of 3D scaffold. The degradation rate of the 300 μm group was slowly than that of the 400 μm group, while the 300 μm group released a higher concentration of bone morphogenesis protein 4 (BMP4) factor than the 400 μm group after 1–7 days of culture. In addition, the expression of SG markers (K18, K19) was detected in the 300 μm group, but not in the 400 μm group within 5 days of culture. These results showed that the pore diameter and geometric parameters of the 3D scaffold printed by the 300 μm printing nozzle were more conducive to cell differentiation and tissue morphogenesis because of the appropriate concentration of nutrition factors and space. When the SG-like tissue was moved to a conventional Petri dish, its morphology disappeared and cells adhered to the bottom of the culture dish, which further proved the importance of geometry (Figure 3) [39].

In general, fabricating patterns of materials at microscale and nanoscale resolutions could regulate cell fate, although it was difficult to mimic the micro-structure of ECM precisely in a 3D environment. There is a need for 3D scaffolds that can be engineered with precise geometric control and micropatterning to mimic the real structural cues.

#### Biochemical and structural cues of 3D-printed scaffold synergistically direct MSC differentiation for functional SG regeneration

**Figure 4.**
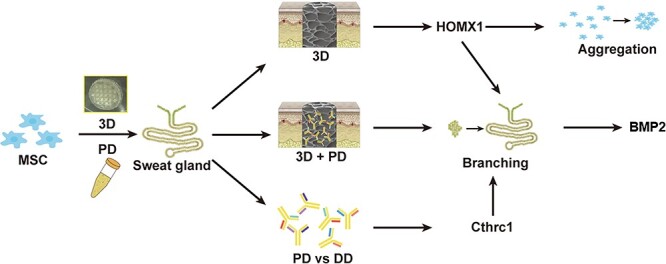
CTHRC1 and *Hmox1* synergistically boost sweat gland fate of MSCs in 3D bioprinted niche. *MSC* mesenchymal stem cell, *DD* dorsal dermis, *Cthrc1* collagen triple helix repeat-containing protein 1, *HOMX1* human heme oxygenase 1, *BMP2* bone morphogenesis protein 2, *PD* plantar dermis

Considering the large demand for seed cells in clinical SG regeneration, we used BMSCs as seed cells for induction in the 3D printed scaffold. Functional SGCs formed 2 weeks later and could specifically repair SGs in mice. Pithelial-mesenchymal transitioin-mesenchymal-epithelial transition (EMT-MET) oscillations were observed during this process, which might have contributed to promoting the transdifferentiation efficiency. In studying the underlying molecular mechanism, it was found through proteomic analysis that collagen triple helix repeat-containing protein 1 (CTHRC1) protein was highly expressed during SG development and acted as a biochemical factor in the 3D scaffold; further transcriptomics analysis proved that 3D structural factors played an indispensable and important role in the differentiation process of BMSCs by up-regulating the expression of Hmox1. Gene ontology functional analysis showed that the 3D structure and soluble factors could synergistically initiate GO items of glandular development and the occurrence of branch structures through activating the expression of BMP2, which is involved in the production of SGs. Adding CTHRC1 antibody to antagonize CTHRC1 protein in biological ink or down-regulating the expression of Hmox1 in BMSC by inhibitors could seriously reduce the inductive effect (Figure 4) [40]. Inflammation factors also inhibited SG differentiation in a 3D construct [41]. In addition to soluble proteins, biochemical factors in the niche, including exosome proteins, non-coding RNA, circulating RNA and circulating cells, were also important to tissue regeneration, and related mechanisms need further study.

#### Vascular niche and SG regeneration

New blood vessel formation in the early stages of wound healing provide nutrients for rapid closure of the wound. A wound cannot close if there are insufficient new blood vessels, resulting in continuous stimulation and chronic wounds such as diabetic wounds [42]. Reestablishment of the vascular system is a prerequisite for the regeneration of appendages after skin transplantation [43].

Some researchers have shown that in addition to providing nutrients and oxygen, blood vessels also regulate the location and behavior of stem cells nearby through cytokine secretion of endothelial cells [44]. In liver, central vein endothelial cells could regulate the self-renewal of peripheral liver cells by secreting Wnt2 and Wnt9b [45]. In various tissues, MSCs located around capillaries differentiated into adipocytes, osteoblasts and smooth muscle cells with factors such as SDF-1, SCF, PDGF-b and Wnt secreted by endothelial cells [46]. Neuronal stem cells differentiated into astrocytes and oligodendrocytes with pigment epithelium-derived factor (PEDF), betacellulin (BTC) and other factors secreted by endothelial cells [47]. Specially, there was abundant horizontal vascular plexus near the activated zone of hair follicle stem cells in telogen, which maintained them in quiescence by secreting BMP4 [44]. In skin, SGs, hair follicles, sebaceous glands and white fat were structurally interrelated to form a skin attachment unit [48]. The fact that hair follicles and SGs had similar development patterns and induction processes [49], as well as the observation that SGs were surrounded by blood vessels, suggested that vascular regulation was essential to the niche of SGs. The anatomical structure of the secretory portion of SGs is located close to blood vessels, and the dermal microvasculature network surrounding several SG units facilitates sweating, however the mechanism of biological communication between vascular endothelial cells and SGCs remains unknown.

#### Neighbor cells and SG regeneration

Adjacent cells and the signals they secrete are indispensable for stem cells to maintain stemness and activation during tissue regeneration [50,51]. Fibroblasts, hair follicles, sebaceous glands and adipocytes are adjacent to SGs and support their morphology and function by secreting Wnt, BMP2, BMP4 and other growth factors. Researchers have shown the degree of cell–cell contact involved in the fate of MSCs or neural stem cells. High cell density or cell proliferation increased cell interactions and promoted maintenance of stemness, while cells at low density were inclined to differentiate because of the weak signals of adjacent cells [52,53].

Fibroblast heterogeneity is responsible for diverse skin functions by providing signals for epidermal differentiation. The dermis is structurally divided into three layers: papillary dermis, reticular dermis and hypodermis. The expression of genes related to the wnt signaling pathway in fibroblasts of the papillary dermis is higher, indicating that it is related to the development and function of skin appendages. The expression of ECM-related genes was higher in the fibroblasts of the reticular dermis, indicating that it was related to wound repair and scar formation. The function of fibroblasts in the hypodermis is related to nutrient supply and fat metabolism as its genes are associated with the adipogenic signaling pathway. Fibroblasts synthesize ECM proteins to form specific structures and regulate the differentiation, stratification and keratinization of the epidermis through secreting soluble factors [54]. Although the subpopulation of fibroblasts responsible for the development of SGs has not been identified, it could control the fate of SGs by artificially manipulating the gene expression of mouse embryonic dermal fibroblasts, which suggested the importance of its role in SG development and function.

#### Mechanical cues and SG regeneration

Changes in the stiffness of the ECM could be transmitted to the nucleus through cell adhesion and the cell cytoskeleton, and then induce the alternation of gene expression [35,55]. BMSCs were used as seed cells to induce SG regeneration while hydrogel stiffness played an extremely important role in the fate of MSCs. They differentiated into adipocytes in hydrogel of 0.1–1 KPa, and into myoblasts or osteoblasts in hydrogels of 1–10 or 10–100 KPa. Muscle stem cells tended to maintain stemness in hydrogels of 1–10 KPa while differentiating into myoblasts in hydrogels of 10–100 KPa. Embryonic stem cells in hydrogels of 0.1–1 KPa differentiated into immature cardiomyocytes, became functionally mature when transferred into 1–10 KPa hydrogel, and even transdifferentiated to fibrotic myofibroblasts in 10–100 KPa hydrogel. In skin, the increase in stiffness of the ECM during wound healing also promotes the transformation of fibroblasts into myofibroblasts, leading to scar [56–58]. Scar firstly changed the composition of dermal fibroblasts, which the adjacent cells of SG damaged, resulted in the lack of signals inducing SG regeneration. Secondly, the increase in ECM stiffness due to accumulation of a large amount of collagen was obstructive for budding and formation of SG ducts which need to penetrate deep into the dermis and are important for sweating. Finally, the increased stiffness of the ECM did not facilitate angiogenesis, which was also harmful to SG regeneration.

## Conclusions

The major challenges of 3D-bioprinted microenvironments for regenerative medicine include how to combine the synergistic cues for accurate cell–matrix signaling and simultaneously deliver a heterogeneous set of cues to recapitulate native microenvironments. For example, by using 3D bioprinting technology and ECM homogenate to mimic SG niche *in vitro* to induce SG regeneration, we proved that structural cues, biochemical factors and mechanical cues were involved in maintaining the function of tissue and driving tissue regeneration. Increased understanding of the key elements involved in 3D bioprinted microenvironments of SG regeneration will likely be important to clarify the difficulties of organ construction *in vitro* and also in advancing the field. We also posit that the facile nature of 3D bioprinting techniques will allow for the creation of vascular and neighbor cellular microenvironments that can robustly and appropriately recapitulate the heterogeneous cellular microenvironment. All of these key elements are typically believed to be essential for successful regeneration of a fully functional organ *in vitro*, and future further exploration of the underlying mechanisms is needed.

## Abbreviations

BMP2: Bone morphogenesis protein 2; BMSC: Bone marrow mesenchymal stem cells; CTHRC1: Collagen triple helix repeat-containing protein 1; ECM: extracellular matrix; EGF; epidermal growth factor; ESC: Epidermal stem cell; SG: Sweat gland; SGC: Sweat gland cell.

## References

[1] Mao AS , Mooney DJ. Regenerative medicine: current therapies and future directions. Proc Natl Acad Sci U S A. 2015;112:14452–9. 26598661 10.1073/pnas.1508520112 PMC4664309

[2] Madl CM , Heilshorn SC, Blau HM. Bioengineering strategies to accelerate stem cell therapeutics. Nature. 2018;557:335–42. 29769665 10.1038/s41586-018-0089-z PMC6773426

[3] Lutolf MP , Gilbert PM, Blau HM. Designing materials to direct stem-cell fate. Nature. 2009;462:433–41. 19940913 10.1038/nature08602 PMC2908011

[4] Lu CP , Polak L, Rocha AS, Pasolli HA, Chen SC, Sharma N, et al. Identification of stem cell populations in sweat glands and ducts reveals roles in homeostasis and wound repair. Cell. 2012;150:136–50. 22770217 10.1016/j.cell.2012.04.045 PMC3423199

[5] Lu C , Fuchs E. Sweat gland progenitors in development, homeostasis, and wound repair. Cold Spring Harb Perspect Med. 2014;4:a015222. 24492848 10.1101/cshperspect.a015222 PMC3904096

[6] Cui CY , Schlessinger D. Eccrine sweat gland development and sweat secretion. Exp Dermatol. 2015;24:644–50. 26014472 10.1111/exd.12773 PMC5508982

[7] Yao B , Xie J, Liu N, Hu T, Song W, Huang S, et al. Direct reprogramming of epidermal cells toward sweat gland-like cells by defined factors. Cell Death Dis. 2019;10:272. 30894517 10.1038/s41419-019-1503-7 PMC6426881

[8] Yao B , Song W, Li Z, Hu T, Wang R, Wang Y, et al. Irf6 directs glandular lineage differentiation of epidermal progenitors and promotes limited sweat gland regeneration in a mouse burn model. Stem Cell Res Ther. 2018;9:179. 29973266 10.1186/s13287-018-0929-7 PMC6033224

[9] Madl CM , Heilshorn SC. Engineering hydrogel microenvironments to recapitulate the stem cell niche. Annu Rev Biomed Eng. 2018;20:21–47. 29220201 10.1146/annurev-bioeng-062117-120954 PMC7266431

[10] Ferraris C , Chevalier G, Favier B, Jahoda CA, Dhouailly D. Adult corneal epithelium basal cells possess the capacity to activate epidermal, pilosebaceous and sweat gland genetic programs in response to embryonic dermal stimuli. Development. 2000;127:5487–95. 11076768 10.1242/dev.127.24.5487

[11] Heinrich MA , Liu W, Jimenez A, Yang J, Akpek A, Liu X, et al. 3D bioprinting: from benches to translational applications. Small. 2019;15:e1805510. 31033203 10.1002/smll.201805510 PMC6752725

[12] Zopf DA , Hollister SJ, Nelson ME, Ohye RG, Green GE. Bioresorbable airway splint created with a three-dimensional printer. N Engl J Med. 2013;368:2043–5. 23697530 10.1056/NEJMc1206319

[13] Laronda MM , Rutz AL, Xiao S, Whelan KA, Duncan FE, Roth EW, et al. A bioprosthetic ovary created using 3D printed microporous scaffolds restores ovarian function in sterilized mice. Nat Commun. 2017;8:15261. 28509899 10.1038/ncomms15261 PMC5440811

[14] An G , Guo F, Liu X, Wang Z, Zhu Y, Fan Y, et al. Functional reconstruction of injured corpus cavernosa using 3D-printed hydrogel scaffolds seeded with HIF-1α-expressing stem cells. Nat Commun. 2020;11:2687. 32483116 10.1038/s41467-020-16192-x PMC7264263

[15] Skardal A , Aleman J, Forsythe S, Rajan S, Murphy S, Devarasetty M, et al. Drug compound screening in single and integrated multi-organoid body-on-a-chip systems. Biofabrication. 2020;12:025017. 32101533 10.1088/1758-5090/ab6d36

[16] Deng K , Ye X, Yang Y, Liu M, Ayyad A, Zhao Y, et al. Evaluation of efficacy and biocompatibility of a new absorbable synthetic substitute as a dural onlay graft in a large animal model. Neurol Res. 2016;38:799–808. 27487559 10.1080/01616412.2016.1214418

[17] Shokouhimehr M , Theus AS, Kamalakar A, Ning L, Cao C, Tomov ML, et al. 3D bioprinted bacteriostatic Hyperelastic bone scaffold for damage-specific bone regeneration. Polymers (Basel). 2021;13:1099. 33808295 10.3390/polym13071099 PMC8036866

[18] Sun H , Hu C, Zhou C, Wu L, Sun J, Zhou X, et al. 3D printing of calcium phosphate scaffolds with controlled release of antibacterial functions for jaw bone repair. Materials & Design. 2020;189:108540.

[19] Vijayavenkataraman S , Yan WC, Lu WF, Wang CH, Fuh J. 3D bioprinting of tissues and organs for regenerative medicine. Adv Drug Deliv Rev. 2018;132:296–332. 29990578 10.1016/j.addr.2018.07.004

[20] Galipeau J , Sensébé L. Mesenchymal stromal cells: clinical challenges and therapeutic opportunities. Cell Stem Cell. 2018;22:824–33. 29859173 10.1016/j.stem.2018.05.004 PMC6434696

[21] Li H , Fu X, Ouyang Y, Cai C, Wang J, Sun T. Adult bone marrow derived mesenchymal stem cells contribute to wound healing of skin appendages. Cell Tissue Res. 2006;326:725–36. 16906419 10.1007/s00441-006-0270-9

[22] Xu Y , Huang S, Ma K, Fu X, Han W, Sheng Z. Promising new potential for mesenchymal stem cells derived from human umbilical cord Wharton’s jelly: sweat gland cell-like differentiative capacity. J Tissue Eng Regen Med. 2012;6:645–54. 21916019 10.1002/term.468

[23] Liang H , Sun Q, Zhen Y, Li F, Xu Y, Liu Y, et al. The differentiation of amniotic fluid stem cells into sweat glandlike cells is enhanced by the presence of sonic hedgehog in the conditioned medium. Exp Dermatol. 2016;25:714–20. 27120089 10.1111/exd.13062

[24] Zhao Z , Xu M, Wu M, Ma K, Sun M, Tian X, et al. Direct reprogramming of human fibroblasts into sweat gland-like cells. Cell Cycle. 2015;14:3498–505. 26566868 10.1080/15384101.2015.1093707 PMC4825576

[25] Blanpain C , Fuchs E. Stem cell plasticity. Plasticity of epithelial stem cells in tissue regeneration. Science. 2014;344:1242281. 24926024 10.1126/science.1242281 PMC4523269

[26] Ozbek S , Balasubramanian PG, Chiquet-Ehrismann R, Tucker RP, Adams JC. The evolution of extracellular matrix. Mol. Biol. Cell. 2010;21:4300–5. 21160071 10.1091/mbc.E10-03-0251 PMC3002383

[27] Yao B , Xie J, Liu N, Yan T, Li Z, Liu Y, et al. Identification of a new sweat gland progenitor population in mice and the role of their niche in tissue development. Biochem Biophys Res Commun. 2016;479:670–5. 27693698 10.1016/j.bbrc.2016.09.155

[28] Murphy SV , Atala A. 3D bioprinting of tissues and organs. Nat Biotechnol. 2014;32:773–85. 25093879 10.1038/nbt.2958

[29] Pati F , Jang J, Ha DH, Won Kim S, Rhie JW, Shim JH, et al. Printing three-dimensional tissue analogues with decellularized extracellular matrix bioink. Nature communication. 2014;5:3935. 10.1038/ncomms4935 PMC4059935 24887553

[30] Lee CH , Rodeo SA, Fortier LA, Lu C, Erisken C, Mao JJ. Protein-releasing polymeric scaffolds induce fibrochondrocytic differentiation of endogenous cells for knee meniscus regeneration in sheep. Sci Trans Med. 2014;6:266ra171. 10.1126/scitranslmed.3009696 PMC4546837 25504882

[31] Grigoryan B , Paulsen SJ, Corbett DC, Sazer DW, Fortin CL, Zaita AJ, et al. Multivascular networks and functional intravascular topologies within biocompatible hydrogels. Science. 2019;364:458–64. 31048486 10.1126/science.aav9750 PMC7769170

[32] Lee A , Hudson AR, Shiwarski DJ, Tashman JW, Hinton TJ, Yerneni S, et al. 3D bioprinting of collagen to rebuild components of the human heart. Science. 2019;365:482–7. 31371612 10.1126/science.aav9051

[33] Shikiji T , Minami M, Inoue T, Hirose K, Oura H, Arase S. Keratinocytes can differentiate into eccrine sweat ducts in vitro: involvement of epidermal growth factor and fetal bovine serum. J Dermatol Sci. 2003;33:141–50. 14643519 10.1016/j.jdermsci.2003.09.004

[34] Huang S , Yao B, Xie J, Fu X. 3D bioprinted extracellular matrix mimics facilitate directed differentiation of epithelial progenitors for sweat gland regeneration. Acta Biomater. 2016;32:170–7. 26747979 10.1016/j.actbio.2015.12.039

[35] Li Y , Kilian KA. Bridging the gap: from 2D cell culture to 3D microengineered extracellular matrices. Adv Healthc Mater. 2015;4:2780–96. 26592366 10.1002/adhm.201500427 PMC4780579

[36] Chen CS , Mrksich M, Huang S, Whitesides GM, Ingber DE. Geometric control of cell life and death. Science. 1997;276:1425–8. 9162012 10.1126/science.276.5317.1425

[37] Roca-Cusachs P , Alcaraz J, Sunyer R, Samitier J, Farré R, Navajas D. Micropatterning of single endothelial cell shape reveals a tight coupling between nuclear volume in G1 and proliferation. Biophys J. 2008;94:4984–95. 18326659 10.1529/biophysj.107.116863 PMC2397343

[38] McBeath R , Pirone DM, Nelson CM, Bhadriraju K, Chen CS. Cell shape, cytoskeletal tension, and RhoA regulate stem cell lineage commitment. Dev Cell. 2004;6:483–95. 15068789 10.1016/s1534-5807(04)00075-9

[39] Liu N , Huang S, Yao B, Xie J, Wu X, Fu X. 3D bioprinting matrices with controlled pore structure and release function guide in vitro self-organization of sweat gland. Sci Rep. 2016;6:34410. 27694985 10.1038/srep34410 PMC5046070

[40] Yao B , Wang R, Wang Y, Zhang Y, Hu T, Song W, et al. Biochemical and structural cues of 3D-printed matrix synergistically direct MSC differentiation for functional sweat gland regeneration. Sci Adv. 2020;6, 10:eaaz1094. 32181358 10.1126/sciadv.aaz1094 PMC7056319

[41] Zhu DZ , Wang YH, Wang R, Fu XB. Effects and mechanism of exogenous tumor necrosis factor α on differentiation of mesenchymal stem cells of mice into sweat gland cells in three-dimensional environment[J]. Chin J Burns. 2020;36:187–94. 10.3760/cma.j.cn501120-20200105-00005 32241044

[42] Veith AP , Henderson K, Spencer A, Sligar AD, Baker AB. Therapeutic strategies for enhancing angiogenesis in wound healing. Adv Drug Deliv Rev. 2019;146:97–125. 30267742 10.1016/j.addr.2018.09.010 PMC6435442

[43] Aki R , Amoh Y, Li L, Katsuoka K, Hoffman RM. Nestin-expressing interfollicular blood vessel network contributes to skin transplant survival and wound healing. J Cell Biochem. 2010;110:80–6. 20225276 10.1002/jcb.22512

[44] Li KN , Jain P, He CH, Eun FC, Kang S, Tumbar T. Skin vasculature and hair follicle cross-talking associated with stem cell activation and tissue homeostasis. Elife. 2019;8:e45977. 31343406 10.7554/eLife.45977 PMC6684267

[45] Wang B , Zhao L, Fish M, Logan CY, Nusse R. Self-renewing diploid Axin2(+) cells fuel homeostatic renewal of the liver. Nature. 2015;524:180–5. 26245375 10.1038/nature14863 PMC4589224

[45] Andreu-Agulló C , Morante-Redolat JM, Delgado AC, Fariñas I. Vascular niche factor PEDF modulates notch-dependent stemness in the adult subependymal zone. Nat Neurosci. 2009;12:1514–23. 19898467 10.1038/nn.2437

[46] Gómez-Gaviro MV , Scott CE, Sesay AK, Matheu A, Booth S, Galichet C, et al. Betacellulin promotes cell proliferation in the neural stem cell niche and stimulates neurogenesis. Proc Natl Acad Sci U S A. 2012;109:1317–22. 22232668 10.1073/pnas.1016199109 PMC3268286

[47] Poblet E , Jimenez F, Escario-Travesedo E, Hardman JA, Hernández-Hernández I, Agudo-Mena JL, et al. Eccrine sweat glands associate with the human hair follicle within a defined compartment of dermal white adipose tissue. Br J Dermatol. 2018;178:1163–72. 29432654 10.1111/bjd.16436

[48] Lu CP , Polak L, Keyes BE, Fuchs E. Spatiotemporal antagonism in mesenchymal-epithelial signaling in sweat versus hair fate decision. Science. 2016;354:aah6102. 28008008 10.1126/science.aah6102 PMC5333576

[50] Ferreira SA , Faull PA, Seymour AJ, Yu TTL, Loaiza S, Auner HW, et al. Neighboring cells override 3D hydrogel matrix cues to drive human MSC quiescence. Biomaterials. 2018;176:13–23. 29852376 10.1016/j.biomaterials.2018.05.032 PMC6011386

[51] Zhang Y , Enhejirigala YB, Li Z, Song W, Li J, et al. Using bioprinting and spheroid culture to create a skin model with sweat glands and hair follicles. Burns Trauma. 2021;9:tkab013. doi: 10.1093/burnst/tkab013. 34213515 PMC8240535

[52] Madl CM , LeSavage BL, Dewi RE, Dinh CB, Stowers RS, Khariton M, et al. Maintenance of neural progenitor cell stemness in 3D hydrogels requires matrix remodelling. Nat Mater. 2017;16:1233–42. 29115291 10.1038/nmat5020 PMC5708569

[53] Darnell M , O'Neil A, Mao A, Gu L, Rubin LL, Mooney DJ. Material microenvironmental properties couple to induce distinct transcriptional programs in mammalian stem cells. Proc Natl Acad Sci U S A. 2018;115:E8368–77. 30120125 10.1073/pnas.1802568115 PMC6130338

[54] Philippeos C , Telerman SB, Oulès B, Pisco AO, Shaw TJ, Elgueta R, et al. Spatial and single-cell transcriptional profiling identifies functionally distinct human dermal fibroblast subpopulations. J Invest Dermatol. 2018;138:811–25. 29391249 10.1016/j.jid.2018.01.016 PMC5869055

[55] Liu Y , Li Z, Li J, Yang S, Zhang Y, Yao B, et al. Stiffness-mediated mesenchymal stem cell fate decision in 3D-bioprinted hydrogels. Burns Trauma. 2020;8:tkaa029. doi: 10.1093/burnst/tkaa029. 32733974 PMC7382973

[56] Vining KH , Mooney DJ. Mechanical forces direct stem cell behaviour in development and regeneration. Nat Rev Mol Cell Biol. 2017;18:728–42. 29115301 10.1038/nrm.2017.108 PMC5803560

[57] Murphy WL , McDevitt TC, Engler AJ. Materials as stem cell regulators. Nat Mater. 2014;13:547–57. 24845994 10.1038/nmat3937 PMC4163547

[58] Smith LR , Cho S, Discher DE. Stem cell differentiation is regulated by extracellular matrix mechanics. Physiology (Bethesda). 2018;33:16–25. 29212889 10.1152/physiol.00026.2017 PMC5866410

